# Verbal Responsiveness in Parents of Toddlers With and Without Autism During a Home Observation

**DOI:** 10.1007/s10803-023-05935-6

**Published:** 2023-05-12

**Authors:** Abigail Delehanty, Jessica L. Hooker, Amy M. Wetherby

**Affiliations:** 1 Department of Speech-Language Pathology, Duquesne University, 600 Forbes Ave., Pittsburgh, PA 15282 USA; 2 Florida State University Autism Institute, 2312 Killearn Center Blvd., Building A, Tallahassee, FL 32309 USA; 3 Department of Clinical Sciences, College of Medicine, Florida State University, Tallahassee, FL 32306-4300 USA

**Keywords:** Autism, Developmental delay, Toddlers, Parent verbal responsiveness, Home observation

## Abstract

This study examined patterns of verbal responsiveness in parents of toddlers (*M*_age_ = 20 months) later identified with autism (*n* = 121), developmental delay (*n* = 46), or typical development (*n* = 44) during an hourlong home observation. Parent verbal responsiveness (PVR) was compared using MANOVA across groups and by child expressive language phase. Multiple regression analyses controlling for child age and maternal education were employed to examine the extent to which PVR predicted variance in concurrent child social communication and prospective language skills. Parents provided synchronous responses approximately 90% of the time. Parents of children with autism and developmental delay used smaller proportions of responses that added linguistic information (i.e., expansions and follow-in directives for language) than those of children with typical development. Parents of children in the preverbal phase were more likely, on average, to affirm their children’s acts of intentional communication or provide a follow-in directive for action that did not necessitate a verbal response than to expand or elicit language. Regression results indicated that parental use of expansions and follow-in directives for language made significant contributions to child language outcomes. The patterns we observed may reflect parents’ attunement to their child’s developmental level. Responsiveness to a child’s focus of attention is vital in the earlier stages of language learning; however, results point to the potential importance of parental expansions and follow-in directives for promoting language development across groups in this sample. Directions for intervention research targeting PVR and language skills in toddlers with autism and developmental delays are discussed.

**Supplementary Information:**

The online version contains supplementary material available at 10.1007/s10803-023-05935-6.

## Introduction

Developmental language disorders are no longer included in the diagnostic criteria for autism spectrum disorder (autism; APA, 2013); however, the two are strongly associated (Casseus et al., 2023; Georginou & Spanoudis, 2021). A significant number of children with autism have difficulties learning to talk (Bishop, 2010; Feurstein et al., 2019). In a transactional model of development, children acquire language in the context of everyday social interactions in many ecological settings with caregivers (McLean & Snyder-McLean, 1978; Sameroff, 1975, 2009). Therefore, studying parent verbal responsiveness (PVR; Edmunds et al., 2019; McDuffie & Yoder, 2010), or talk that “follows in” to a child’s focus of attention or communicative acts, has long been a topic of interest for researchers studying children with and without developmental delays (DD) and autism (Brady et al., 2004; Hart & Risley, 1978; Siller & Sigman, 2002; Tamis-LeMonda et al., 2001). A number of developmental studies has been published as researchers seek to increase understanding of how parents attune their language based upon their child’s developmental profile and to identify the features of PVR that positively impact social communication and language development (e.g., Choi et al, 2020; Dimitrova et al., 2016; Jokihaka et al., 2022; Venker et al., 2012). Further, several parent-implemented early intervention models for children with autism and DD include supporting caregiver-child interaction and increasing PVR as core components (e.g., Brian et al., 2022; Carter et al., 2011; Green et al., 2010; Heidlage et al., 2020; Watson et al., 2017; Wetherby et al., 2014; WHO, 2022). The purpose of this study was to examine patterns of verbal responsiveness to child communicative acts in a sample of parents of toddlers later identified with autism, DD, or typical development (TD) during a naturalistic home observation.

## Parent Verbal Responsiveness and Children with Typical Development

For children with TD, a large body of research supports significant relations between maternal sensitivity, a broad concept encompassing a mother’s ability to respond contingently, predictably, and warmly to infant signals, emotions, and behaviors, and child language development (Tarabulsy et al., 2016). In long-term follow-up studies, for instance, maternal sensitive responses in the first 3 years of life were significantly associated with child social competence and academic achievement at multiple time points from middle childhood to adulthood (Fraley et al., 2013; Raby et al., 2015). Perhaps most strikingly, studies of maternal sensitivity from developed and developing countries have found significant relationships with not only social and cognitive outcomes, but also reductions in child disease and mortality (Eshel et al., 2006).

Research findings related to PVR, a subcategory of parental sensitivity, indicate that language acquisition is promoted when children hear words that map directly onto the object or event to which they are attending and about which they are communicating (Gros-Louis et al., 2014; Masek et al., 2021; Tomasello & Farrar, 1986). PVR is associated with the achievement of critical language milestones developing in the second year, including the onset of first words, first 50 words, word combinations, and grammatical development (Lopez et al., 2020; Rollins & Snow, 1998; Tamis-LeMonda et al., 2001). As children’s initiations become clearer and more frequent, their parents in turn respond with increased complexity, thus scaffolding the child’s developing skills (Rowe & Snow, 2020; Wetherby et al., 1998). Therefore, parental expansions, which include the child’s words while also adding semantic or grammatical information, are particularly strong predictors of later language skill (Bornstein et al., 2008; Taumoepeau, 2016). Taken together, these findings provide strong support of PVR for typical language and communication development and have inspired a number of studies of PVR in children with DD and autism.

## Parent Verbal Responsiveness and Children with Developmental Delays and Autism

Children with autism and DD frequently evidence communication and language delays. Early in the developmental period, toddlers with autism have been observed to communicate at significantly lower rates per minute and spend a lesser amount of time in coordinated joint engagement with their parents compared to those with TD (Adamson et al., 2019; Delehanty & Wetherby, 2021; Roemer et al., 2022). Research also indicates that young children with DD communicate less frequently than children with TD (e.g., Delehanty et al., 2018; Slonims & McConachie, 2006). With fewer chances for parents to respond contingently to their child’s communicative acts, opportunities for social interaction and language learning may be limited. Thus, cascading effects on both frequency and quality of parent–child interactions may occur. Even still, research results indicate that PVR is related to language development in children with DD and autism (Bottema-Beutel & Kim, 2021; Edmunds et al., 2019; Wan et al., 2019).

In the earliest published studies in this area, Siller and Sigman (2002, 2008) examined how parents of children with autism, DD, and TD, followed their child’s focus of attention. Parents of children in all three groups used a statistically equivalent proportion of “synchronous” PVR, or talk that followed the child’s focus of attention and was undemanding with respect to asking the child to change their attentional focus or behavior. For the present study, this type of synchronous PVR is termed *follow-in commenting*. Proportional increases of parental follow-in comments were found to be associated with long-term gains in children’s communication and language skills (Siller & Sigman, 2008). In a study of younger children, Dimitrova et al. (2016) examined PVR in dyads that included children with autism, Down Syndrome, and TD. They also found that mothers of children with autism were as likely as mothers of children with TD and Down Syndrome to be highly responsive to their children’s gestures, and that linguistic mapping, a type of follow-in comment that provided a “translation” of child gesture into words using an explicit label (Edmunds et al., 2019; Yoder & Warren, 2002), was related to increases in expressive vocabulary. Thus, existing evidence suggests that parental follow-in comments are positively related to language development in children with autism and DD.

Rather than responding to their child’s communicative acts with follow-in comments like linguistic mapping and expansions, some parents adopt a more directive communication style, refocusing their child’s attention or requesting that they change their actions (Freeman & Kasari, 2013; Wan et al., 2012; 2013). Directive parental communication has been associated with fewer child gestures and prelinguistic vocalizations in infants and toddlers with TD (Miller & Gros-Louis, 2013). However, young children with DD and autism have difficulty shifting attention between objects and people as well as initiating and maintaining a joint attentional focus (Adamson et al., 2019; Delehanty et al., 2018). They may benefit from at least some directive, follow-in language to support language and prolonged engagement in the interaction. For the present study, this PVR type is termed *follow-in directing* (McDuffie & Yoder, 2010).

In a study examining the associations between PVR and vocabulary development in children with autism, McDuffie and Yoder (2010) examined parents’ use of follow-in comments and directives. Use of both response types was significantly associated with parent-reported expressive vocabulary 6 months later, and parental expansions contributed a small but significant amount of unique variance to predicting change in vocabulary. Another pair of studies expanded the work of McDuffie and Yoder (2010) by examining follow-in directives for language and action separately (Haebig et al., 2013a, 2013b). Follow-in directives for language were found to have significant, positive associations with child language 1 and 3 years later for their sample of children with autism. Haebig and colleagues (2013a; 2013b) also explored PVR in a subgroup of children who used fewer than five words during their initial evaluation session. They found that parental use of follow-in comments accounted for unique variance in later language for minimally speaking children, but not for those using more language. The authors speculated that children who used more words would benefit from parental input that included more developmentally enhanced language forms.

The growing research base in this area has motivated three recent systematic literature reviews examining associations between PVR and language skills in children with autism (Bottema-Beutel & Kim, 2021; Edmunds et al., 2019; Wan et al., 2019). Most studies reviewed generally indicated that linguistic mapping, expansions, and follow-in directives were predictive of later language in children with autism. The authors of these reviews also uncovered gaps in the literature and recommended directions for future research on PVR. Among these were studies with well-defined links to theoretical frameworks of language development, clearer operationalization of PVR, the inclusion of both responsive and directive PVR, and larger sample sizes of children at different ages and developmental stages. Specifically, Edmunds and colleagues (2019) found that around half of the 25 studies included in their review included participants older than 3 years of age, making difficult to form conclusions about younger children who may be actively learning single words as well as acquiring more complex forms. Next, only a small number of published studies to date has examined PVR in samples with comparison groups of young children with autism, DD, and TD (Dimitrova et al., 2016; Siller & Sigman, 2002, 2008) or explored variation in PVR by child expressive language level (Haebig et al., 2013a, 2013b;). Given evidence that children with DD and autism both show less robust language and communication skills compared to their peers with TD, examining patterns of PVR in both diagnostic groups may add important information to the literature base in this area. Finally, few studies of PVR have used observational coding in the home environment (e.g., Leezenbaum et al., 2014). Both Bottema-Beutel and Kim (2021) and Wan et al. (2019) recommended research that characterizes PVR in natural social contexts that include a range of family activities in order to add information to studies that have examined PVR in laboratory settings.

## Purpose of the Current Study

The purpose of this study was to examine patterns of verbal responsiveness to child communicative acts in a sample of 211 parents of toddlers later identified with autism, DD, or TD who interacted during an hourlong, video-recorded observation in the home environment. This study was guided by three aims, with hypotheses that were both theoretically and empirically motivated. First, we aimed to describe and compare patterns of PVR across diagnostic groups. Following previous work in this area, we hypothesized that parents in all groups would provide PVR that followed their child’s focus of attention a large proportion of the time (e.g., Dimitrova et al., 2016; Pijl et al., 2021). Based on observations that parents of children with autism may use a more directive communication style (e.g., Wan et al., 2019), we also anticipated that parents of children with autism would use significantly more follow-in directives than those of children with TD.

Our second aim was to explore PVR across subgroups of children classified according to their expressive language phase. Results of studies of children with and without autism indicate that PVR may vary with the child’s expressive language level (e.g., Haebig et al., 2013a, 2013b; Vallotton et al., 2017). From a transactional framework, we hypothesized that parents of children who used clearer, more sophisticated child communicative acts would be more likely to imitate and add linguistic information to their children’s utterances. Therefore, we anticipated that parents of children using words and word combinations would use larger proportions of follow-in comments than those of children who communicated using gestures and sounds.

Third, we aimed to examine the extent to which PVR predicted variance in concurrent social communication and prospective expressive and receptive language skills. Research results have indicated that PVR that is responsive to the child’s focus of attention, particularly the use of linguistic mapping, expansions, and follow-in directives, is associated with increased child engagement and language learning (Bottema-Beutel & Kim, 2021; Edmunds et al., 2019; Wan et al., 2019). Therefore, we predicted that parents’ use of these PVR types would be positively associated with concurrent social communication and prospective receptive and expressive language skills. Studies examining concurrent and prospective associations between PVR, child social communication, and language outcomes are limited. This study also extends earlier work by examining PVR in a large sample of parents and younger toddlers that includes comparison groups of children with autism, DD, and TD, grouped by expressive language level, interacting in a systematic yet naturalistic home observation.

## Methods

### Participants

Families were participants in the FIRST WORDS Project, a longitudinal research investigation that aims to identify early signs of communication disorders and autism in children 9–24 months of age (Wetherby et al., 2008). At a mean age of 19.4 months (*SD* = 2.1), children in the current study completed a communication evaluation that included a video-recorded Communication and Symbolic Behavior Scales Behavior Sample (CSBS; Wetherby & Prizant, 2002). An hourlong home observation (*M* = 56.1 min, *SD* = 6.3) was conducted at 20.3 months (*SD* = 2.0). At 36.6 months (*SD* = 4.8), a clinical best estimate diagnosis was made at a developmental evaluation using all available information by an experienced team of diagnosticians including a licensed psychologist, speech-language pathologist, and early childhood specialist (Kim & Lord, 2012). All children completed the Mullen Scales of Early Learning (MSEL; Mullen, 1995) as part of the developmental evaluation. Children who were identified with DD had *T* scores of at least 1.25 *SD* below the mean on any subscale of the MSEL. All children with DD and those with concerns about autism also completed the Autism Diagnostic Observation Schedule (ADOS; Lord et al., 1999). Children were identified as TD if they scored within 1.25 *SD* of the mean or higher on all MSEL scales and there were no concerns about autism. Diagnostic outcomes and other developmental characteristics of the children in this sample were reported in Delehanty and Wetherby (2021) for all children included in this study and are included in Supplemental Table S1.

The larger project oversampled male children to recruit a pool to match the group of children with autism on sex; therefore, 81% of child participants were male. Most participants in the study identified as white (68%), followed by Black (20%), more than one race (10%), and Asian (2%). Approximately 8% of participants reported Hispanic ethnicity. About 23% of mothers graduated from high school, 22% graduated from college, and 27% had a graduate degree. Among fathers, 31% completed high school, 17% had a college degree, and 23% had a graduate degree. On average, mothers were 31 years old at the time of their child’s birth, and fathers were 33 years old. Additional demographic information is included in Supplementary Table S2. All parents gave written informed consent. This study was approved by the Institutional Review Board at Florida State University.

## Measures

### Communication and Symbolic Behavior Scales Behavior Sample (CSBS)

The CSBS is a standardized observational measure of communication for use with children from 12 to 24 months, and yields three composite scores (*M* = 10, *SD* = 3) as well as a total score (*M* = 100, *SD* = 15). The social composite measures expression of affect, use of eye gaze, communication, and gestures. The speech composite measures use of sounds and words. The symbolic composite measures language comprehension and object use in play.

### Mullen Scales of Early Learning (MSEL)

The MSEL is a standardized cognitive assessment appropriate for children from 1 to 68 months. *T* scores may be calculated for receptive language (RL), expressive language (EL), visual reception (VR), and fine motor (FM) scales (*M* = 50, *SD* = 10). The Early Learning Composite has a mean of 100 (*SD* = 15).

## Coding Scheme for the Home Observation

The purpose of the baseline home observation was to collect information about child communication, social interaction, and play during everyday activities with a caregiver or caregivers. Families received a set of standardized instructions in advance of the recording, which were reviewed by a member of the research team prior to conducting the observation. Families were asked to interact with their child while participating in as many of the following activities as possible: play with toys, play with people, meals or snacks, caregiving, book sharing, and family chores. One adult interacted with the child in 61% of home observation videos. Fathers were the primary communication partner in 3% of videos. Noldus Pro^©^ Observer XT v12.5 software was utilitzed for coding.

### Child Communicative Acts

Delehanty and Wetherby (2021, 2022) reported the results of coding over 40,000 child communicative acts for this sample. Using criteria from the CSBS, child communicative acts were coded as mutually exclusive and exhaustive behaviors that: (a) included a deictic or representational gesture, sound (i.e., nonword vocalization), word, word combination, or temporally overlapping gesture + sound, word, or word combination; (b) were directed toward the adult; and (c) served a communicative function (Wetherby & Prizant, 2002). The quality of the home video footage varied; therefore, child eye gaze to faces was not coded. Following criteria outlined by Wetherby and Prizant (2002), each communicative act was determined to be directed toward an adult if the child communicated while looking at or turning to face the adult, moving self or an object toward the adult, or using coordinated verbal and nonverbal communication (e.g., gesture + word). Interrater reliability for identification of child communicative acts by type and function was calculated and results indicated acceptable agreement (*κ* = .80–.84; Cohen, 1960; McHugh, 2012).

### Parent Verbal Responsiveness

For the current study, following previous work in this area (Cress et al., 2013; Haebig et al., 2013a; Tamis-LeMonda et al., 2013), coders examined the three seconds (< 4 s) after each child communicative act to determine whether a contingent parent verbal response occurred. Each contingent response was identified and coded as synchronous or asynchronous based on whether the parent referenced the same entity or event that the child was focused on. Modifiers were assigned to further characterize each parent response. Coding definitions are presented in Table 1. The entire coding scheme is available by request.

**Table 1:** Coding scheme for parent verbal responsiveness

Synchronous	Parent responds to the child’s communicative act within 3 s, and the response follows the child’s focus of attention
Follow-in verbal comment	Parent comments but does not ask the child to do or say anything
Linguistic mapping	Parent provides the word the child approximated or a word for an object or event the child seems to be referencing, without adding words
Attribute	Parent describes an object or action that was the focus of the child’s communicative act, but a label is not provided (e.g., “It says quack!”)
Expansion	Child uses a word approximation, single word, or phrase. Parent repeats what the child says and adds additional information
Follow-in directive	Parent asks that the child do or say something in relation to what the child is focused on. The child does not have to subsequently do or say anything for this response to be coded
For language	Parent directs the child to communicate about something the child is already focused on (e.g., invites the child to imitate a word, asks a responsive question that necessitates a verbal response)
For action	Parent directs the child to do something during an ongoing activity in which the child is engaged
Follow-in nonverbal comment	Parent follows the child’s focus of attention by responding with a complementary or imitative gesture or an affirming utterance that does not add linguistic information (e.g., “Uh oh!” “Mm-hm,” “Okay,” “Thank you,” “Wow,” sound effects, etc.)
Asynchronous	Parent responds within 3 s, but the content of the utterance is unrelated to the child’s focus of attention
Asynchronous comment	Parent responds with a comment about something that does not follow the child’s focus of attention, but does not ask the child to do or say anything
Asynchronous directive	Parent asks the child to do or say something that redirects their focus of attention or changes their actions
Missed Opportunity	Parent makes no response to the child, or the response had a latency of greater than 3 s

Modifiers to synchronous PVR included follow-in verbal comments, follow-in directives, and follow-in nonverbal comments. Follow-in verbal comments included: a) linguistic mapping, in which the parent provided an explicit label for a word the child seemed to be trying to say, without adding information (e.g., the child references a duck and says, “uh!” The parent says, “Duck!”); b) attributes, where the parent described an object or action that was the focus of the child’s communicative act, but did not provide an explicit label (e.g., “It says quack!”); and c) expansions, whereby the parent repeated what the child said and added linguistic information (e.g., Child says, “Duck.” Parent responds, “Duck! The duck is swimming.”). Follow-in directives were coded for language or action. Follow-in directives for language were coded when the parent invited the child to communicate about something they were focused on (e.g., asking the child to imitate a word or asking a question that necessitated a verbal reply). Follow-in directives for action were assigned when the parent directed the child to do something related to an ongoing activity (e.g., Let’s put the duck in the water.”). Follow-in nonverbal comments were defined as synchronous affirmative tokens (McDuffie & Yoder, 2010) that did not add linguistic information to the child’s communicative act (e.g., “Mm-hm,” “Okay,” “Wow,” providing sound effects, etc.). Asynchronous comments and directives were coded when the parent’s response was unrelated to the child’s focus of attention. Finally, if the parent did not respond to the child’s communicative act within 3 s or spoke to another person in the room instead, this was coded as a missed opportunity. If the parent’s response was inaudible, it was considered uncodeable (*M* = 1% of all PVR, *SD* = 4.0).

## Training and Interrater Reliability

Observational coders for this study were trained undergraduate research assistants, unaware of hypotheses and participant diagnoses. We reviewed coding procedures and definitions and practiced consensus coding of home observations not included in this study across one academic semester. Ten training videos of dyads that included children from all three diagnostic groups (not included in this study) were selected, and inter-rater reliability in the form of point-by-point agreement was assessed using Cohen’s kappa coefficients (Cohen, 1960; McHugh, 2012). An agreement criterion of *κ* > .60 (Landis & Koch, 1977) was set for the identification of the PVR type that occurred most proximal to the child’s communicative act. Only one parent behavior was coded per child communicative act. There were instances in which the parent produced a follow-in nonverbal comment and followed up with an utterance that added new linguistic information. In these cases, the second response was coded. For example, if the child said, “juice,” and the parent responded with a follow-in nonverbal comment (e.g., “Okay.”) then subsequently used an expansion (e.g., “More apple juice!”), we coded the expansion. Forty-two videos (20%) were independently double-coded. The κ for coding the type of parent response was .77, 95% CI [.76, .78], indicating substantial agreement.

## Analytic Plan

### Aim 1. Patterns of PVR Across Groups

We compared proportions of PVR types using one-way multivariate analysis of covariance (MANCOVA; Group × PVR), controlling for baseline child expressive language (CSBS speech composite score). Statistically significant results were followed up with Bonferroni-corrected post hoc, pairwise comparisons.

### Aim 2. Exploring PVR by Language Phase

We characterized each child’s language phase using expressive language benchmarks developed by experts assembled by the National Institute on Deafness and Other Communication Disorders (Tager-Flusberg et al., 2009). These benchmarks use a developmental framework to provide uniform terminology for describing expressive language in children with autism and have proved to be useful for investigating variables related to children’s language growth (Ellawadi & Weismer, 2015; Trembath et al., 2022). The guidelines of Tager-Flusberg and colleagues (2009) denote that language phases may be determined using multiple sources including language samples collected during administration of semi-structured direct assessment measures. For the current study, following the outlined minimum criteria for classification, the preverbal phase was defined as an inventory of 0–1 words expressed during the CSBS Behavior Sample. The early first words phase was assigned if the child used 2–5 different non-imitated, spontaneous, intelligible single words during the CSBS. Children in the late first words phase used 6–16 different single words (the highest number of different words counted during the CSBS Behavior sample) and 0–1 different word combinations. Finally, children in the word combinations phase used 10 or more single words, two or more creative word combinations, and a variety of communicative functions.

**Fig. 1:**
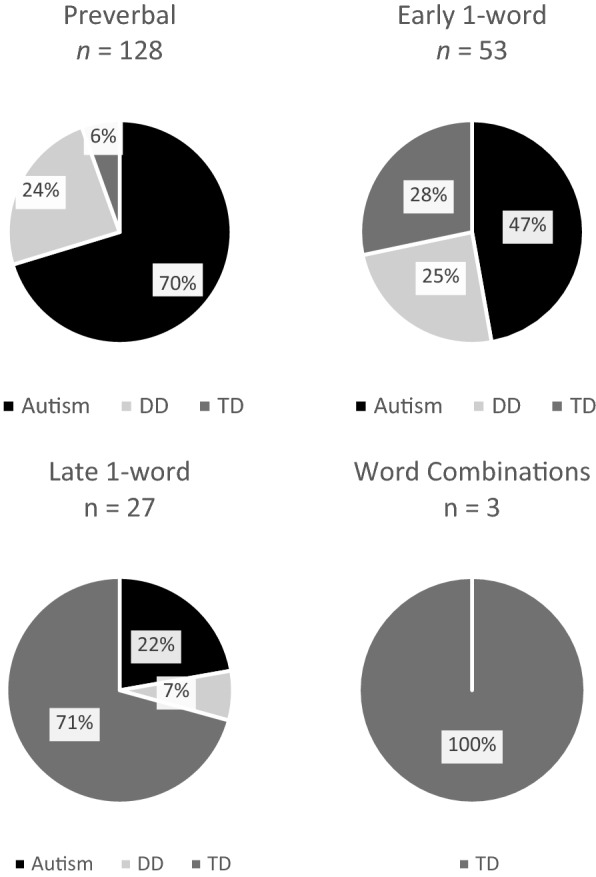
Expressive language phases across diagnostic groups. *DD* Developmental delay without autism, *TD* Typical development, *N* = 211. The sample size for diagnostic groups was *n*_autism_ = 121, *n*_DD_= 46, *n*_TD_ = 44

The distribution of children from our three diagnostic groups by language phase is displayed in Fig. 1. Only three children, all in the typically developing group, met minimum criteria for the word combinations phase; therefore, we combined children in the late first words and word combinations phases into one group. Proportions of PVR were compared using one-way MANOVA (language phase × PVR), and statistically significant results were followed up with Bonferroni-corrected post hoc, pairwise comparisons.

### Aim 3. Concurrent and Prospective Relationships

We calculated Pearson product-moment correlation coefficients to examine linear relationships among variables, adjusted using a Bonferroni correction due to the large number of associations examined. Bivariate correlations guided the hierarchical multiple regression analyses, which tested whether selected PVR variables significantly predicted concurrent social communication (CSBS total score) and prospective receptive and expressive language (MSEL verbal developmental quotient score, where DQ = [mean of RL and EL age equivalent scores/chronological age] × 100). DQ was determined to be appropriate to encapsulate the variability of the children in our sample, as many children scored below the basal on the MSEL raw score (Kim and Lord, 2012). A large, significant correlation was observed between RL and EL DQ scores (*r* = 0.86, *p* < 0.001). Therefore, the verbal DQ was selected for analyses.

Average proportions of PVR types were used in all analyses to control for influences of child base rate of communication. Child age and maternal education level were included as covariates in each model, given significant group differences in our sample and following previous work in this area (e.g., Jokihaka et al., 2022; Pecukonis et al., 2022). We also controlled for child baseline expressive language (CSBS speech composite) in our examination of prospective relationships. We calculated *f*^2^ to estimate effect size, where *f*^2^ = *R*^2^/(1−*R*^2^) with an *f*^*2*^ of .02 = small effect, medium *f*^*2*^ = .15, and large *f*^*2*^ = .35 (Cohen, 1988).

## Results

### Patterns of PVR Across Diagnostic Groups

Proportions of PVR used during the home observation are displayed in Table 2. Approximately 90% of PVR was synchronous for the entire sample. A statistically significant difference in PVR was observed based on diagnostic group, *F*(16, 400) = 1.88, *p* = 0.02; Wilk’s Λ = 0.865, partial η^2^ = 0.07. Parents of children with TD used a significantly larger proportion of synchronous PVR (*M* = .93, *SD* = .06) than those of children with autism (*M* = .89, *SD* = .11) and DD (*M* = .87, *SD* = .13), *F*(2, 208) = 3.78, *p* < .05. Autism and DD groups did not differ.

**Table 2:** Proportions of parent verbal responsiveness across diagnostic groups

Diagnostic Group	Autism		DD		TD				
	(*n* = 121)		(*n* = 46)		(*n* = 44)				
Proportions	*M*	*SD*	*M*	*SD*	*M*	*SD*	*F* (2, 207)	η2p	Post-hoc pairwise comparisons (Bonferroni *p *< .05)
Synchronous									
Follow-in Verbal Comment									
Linguistic Mapping	.11	.09	.11	09	.17	.09	0.64	.01	
Attribute	.05	.05	.05	.05	.08	.06	2.82	.03	
Expansion	.01	.02	.01	.01	.03	.02	5.20**	.05	ASD, DD < TD
Follow-in Directive									
For Language	.16	.09	.14	.08	.21	.06	3.42*	.04	ASD, DD < TD
For Action	.16	.09	.16	.08	.13	.06	0.60	.01	
Follow-in nonverbal comment	.39	.15	.39	.16	.32	.13	0.32	<.01	
Asynchronous									
Comment	<.01	.01	.01	.02	<.01	.01	2.29	.02	
Directive	.02	.03	.03	.04	.01	.02	1.78	.02	
Missed opportunity	.10	.11	.10	.11	.05	.05	2.71	.03	

Proportions represent total count/all parent verbal responses

*DD* Developmental delay without autism, *TD* Typical development, *η*^*2*^*p* .01 is interpreted as a small effect, .06 = medium effect, .14 = large effect (Cohen, 1988)

*p < .05, **p < .01 derived using MANCOVA controlling for child expressive language (Communication and Symbolic Behavior Scales speech composite)

Examining each type of synchronous PVR, parents of children in the TD group used a significantly larger proportion of expansions than those of children with autism and DD, with small effect sizes (Mean difference ± SE = .02 ± .01, *p* < .001, 95% CI [.01, .03]). Parents of children with TD also used follow-in directives for language in significantly larger proportions than those of children with autism and DD, again with small effect sizes (Mean difference ± SE for children with autism = .05 ± .01, *p* < .01, 95% CI [.01, .08]; DD = .06 ± .02, *p* < .01, 95% CI [.02, .11]). Parents of children autism and DD were not significantly different on their use of these response types. We did not observe significant group differences for parental use of follow-in nonverbal comments, asynchronous responses, or missed opportunities.

### Exploring PVR Across Child Language Phases

Turning to our second aim, we observed a statistically significant difference in PVR based on child expressive language phase, *F*(18, 400) = 5.32, *p* < .001; Wilk's Λ = 0.651, partial η^2^ = .19 (Table 3). Beginning with follow-in verbal comments, parents of children in all three phases differed from one another on the proportions of linguistic mapping and expansions used, with large effect sizes (Mean difference ± SE for linguistic mapping: pre < early = .06 ± .14, *p* < .001, 95% CI [.03, .09]; pre < late = .12 ± .02, *p* < .001, 95% CI [.08, .16]; early < late = .06 ± .02, *p* < .01, 95% CI [.01, .10]; Expansions: pre < early = .01 ± .003, *p* < .01, 95% CI [.003, .01]; pre < late = .03 ± .004, *p* < .001, 95% CI [.02, .04]; early < late = .02 ± .004, *p* < .01, 95% CI [.01, .03]).

**Table 3:** Proportions of parent verbal responsiveness by child expressive language phase

Language Phase	Preverbal		Early First Words		Late First Words/Word Combinations				
	(*n* = 128)		(*n* = 53)		(*n* = 30)				
Proportions	*M*	*SD*	*M*	*SD*	*M*	*SD*	*F* (2, 208)	η^2^p	Post-hoc pairwise comparisons (Bonferroni *p* < .05)
Synchronous									
Follow-in Verbal Comment									
Linguistic Mapping	.09	.07	.15	.08	.21	.11	30.15***	.23	Pre < early; pre, early < late
Attribute	.06	.05	.06	.05	.07	.05	0.54	.01	
Expansion	.01	.01	.02	.02	.03	.02	26.68***	.20	Pre < early; pre, early < late
Follow-in Directive									
For Language	.15	.09	.18	.08	.20	.08	4.98**	.05	Pre < late
For Action	.17	.09	.15	.07	.11	.06	5.03**	.05	Pre > late
Follow-in Nonverbal Comment	.41	.16	.33	.11	.30	.14	10.47***	.09	Pre > early, late
Asynchronous									
Comment	.01	.01	.01	.01	.01	.01	0.74	.01	
Directive	.02	.03	.02	.04	.01	.01	0.50	.01	
Missed Opportunity	.08	.09	.07	.10	.05	.05	0.77	.01	

Proportions represent total count/all parent verbal responses

*η*^*2*^*p* .01 is interpreted as a small effect, .06 = medium effect, .14 = large effect (Cohen, 1988)

*p < .05, **p < .01, ***p < .001 derived using one-way MANOVA

With regard to follow-in directives, parents of children in the preverbal phase used a smaller proportion of follow-in directives for language and a larger proportion of follow-in directives for action than those of children in the late first words stage (Mean difference ± SE for follow-in directives for language = .05 ± .02, *p* < .05, 95% CI [.01, .09]; follow-in directives for action = .05 ± .02, *p* < .01, 95% CI [.01, .10]), with medium and small effect sizes, respectively. For follow-in nonverbal comments, parents of children in the preverbal phase used a significantly larger proportion of this PVR type than those of children in the early and late first words phases, with a medium effect size (Mean difference ± SE for pre > early = .08 ± .02, *p* < .01, 95% CI [.03, .14]; pre > late = .11 ± .03, *p* = .001, 95% CI [.04, .18]). Echoing our findings for PVR by diagnostic group, parents did not differ on the proportions of asynchronous responses and missed opportunities used.

### Concurrent and Prospective Relationships

Bivariate correlations between PVR and the CSBS total score and MSEL verbal DQ are reported in Table 4. Three parent response variables that yielded significant zero-order concurrent correlations with the CSBS and prospective correlations with the MSEL after Bonferroni correction were selected as predictors in the hierarchical multiple regression analyses: linguistic mapping, expansions, and follow-in directives for language. Results of the first regression indicated that child age and maternal education level accounted for 11% of the variance in the CSBS total score with a small effect size, *F*(2, 208) = 11.36, *p* < .001, *f*^2^ = .12. Maternal education contributed significantly to the model (B = 2.84, *p* < .001) while child age did not (B = − 0.91, *p* = .09). The next model that included our PVR variables explained 30% of the variance and was a significant predictor of the CSBS total score with a large effect size, *F*(3, 205) = 16.31, *p* < .001, *f*^2^ = .43. Proportions of parental linguistic mapping, expansions, and follow-in directives for language each contributed significantly to the model (B = 46.97, *p* < .001, B = 170.68, *p* < .001, and B = 36.19, *p* < .001, respectively).

**Table 4:** Bivariate correlations between parent verbal responsiveness and measures of child social communication and language

Parent Verbal Response (Proportions)	CSBS	MSEL
	Total Score^a^	Verbal DQ^b^
Synchronous		
Follow-in Verbal Comment		
Linguistic Mapping	.40*	.38*
Attribute	.12	.15
Expansion	.40*	.37*
Follow-in Directive		
For Language	.23*	.28*
For Action	− .19	− .22*
Follow-in Nonverbal Comment	− .28*	− .27*
Asynchronous		
Comment	.01	− .04
Directive	− .04	− .09
Missed Opportunity	− .11	− .12

Proportions represent total count/all parent verbal responses

*CSBS* Communication and Symbolic Behavior Scales Behavior Sample, administered at 20 months. *MSEL* Mullen Scales of Early Learning, administered at age 3

*Bonferroni-corrected *p* < .001

^a^Standard Scores based on *M* of 100 and *SD* of 50 were used in analyses

^b^DQ = Development Quotient was used in analyses

Turning to prospective associations with language skills, the first model including maternal education, child age, and child baseline expressive language explained 36% of the variance in MSEL verbal DQ and was statistically significant with a large effect size, *F*(2, 208) = 36.12, *p* < .001, *f*^2^ = .56. All three variables contributed significantly to the model (maternal education: B = 4.52, *p* < .001; child age: B = −2.48, *p* = .04; and child expressive language: B = 7.51, *p* < .001). The second model including PVR explained 42% of the variance and was statistically significant with a large effect size, *F*(3, 205) = 22.32, *p* < .001, *f*^2^ = .72. Parents’ use of expansions (B = 205.76, *p* < .001) and follow-in directives for language (B = 40.79, *p* = .04) contributed significantly to the model, while linguistic mapping did not (B = 39.11, *p* = .07).

## Discussion

The overarching goal of this study was to examine patterns of verbal responsiveness in parents of toddlers observed during everyday activities at home. This study expands on previous work by using granular observational coding of parent–child interaction during a naturalistic, hourlong home observation in a large sample of toddlers that included comparison groups of children with autism, DD, and TD who were also grouped by expressive language phase. We then examined associations between PVR, concurrent child social communication, and prospective receptive and expressive language. Overall, parents were exceedingly responsive to their children’s communicative acts. Differences in PVR were more readily apparent when children were classified according to expressive language phase than by diagnostic group. Parental expansions and follow-in directives for language were observed to contribute significantly to child language outcomes.

### PVR by Diagnostic Group and Language Phase

For our first aim comparing patterns of PVR across diagnostic groups, our hypotheses were partially supported. On average, parents in all three groups provided contingent, synchronous responses about 90% of the time, a finding that supports previous research (e.g., Choi et al., 2020; Dimitrova et al., 2016; Siller & Sigman, 2002). We add to the literature with our finding that this pattern of overwhelmingly synchronous PVR was demonstrated during a naturalistic, home-based observation of approximately an hour’s time. We controlled for child expressive language in our analyses, anticipating that this variable would explain differences in PVR across our diagnostic groups. Parents of children in the TD group were observed to use a significantly larger proportion of expansions than parents of children with autism and DD. They also used a larger proportion of follow-in directives for language, which was unexpected given that we anticipated parents of children with autism would evidence a more directive communication style. No other significant differences were detected. Moreover, parents of children with autism and DD did not differ with respect to their use of any PVR type.

These findings underscore the importance of considering variation in child language level, our second aim, in addition to studying differences in patterns of PVR by diagnostic group. We observed key differences in PVR based on child expressive language phase that supported our hypotheses. Parents of children in the late first words/word combinations phase used significantly larger proportions of linguistic mapping and expansions than those of children in the earlier language phases. They also used more follow-in directives for language and fewer follow-in nonverbal comments and follow-in directives for action than those of preverbal children. Thus, parents of children in the preverbal phase were more likely, on average, to affirm their children’s acts of intentional communication or give a direction that did not necessitate a verbal response than to expand or elicit additional linguistic information.

Examining CSBS and MSEL scores (Supplemental Table S1) as well as the representation of children across language phases (Fig. 1) revealed that children in the TD group used communicative acts that were more sophisticated in form than those used by most children with autism and DD. Therefore, it is conceivable that parents of children in the later phases could easily decipher more of their children’s messages and respond by offering new linguistic information. Children with autism in this sample scored significantly lower than DD and TD groups on the CSBS social composite, largely due to their reduced rate per minute of communication and restricted inventory of communicative gestures. However, children with autism and DD did not differ significantly from one another on the CSBS speech and symbolic composites or MSEL expressive and receptive language T scores. Therefore, the patterns of PVR we observed appear to reflect parents’ attunement to their child’s language above and beyond their social communication and social interaction skills. A lower rate of child communicative acts may reduce the number of opportunities for providing PVR; however, findings indicated that parents were responsive to the quality of their child’s speech and language notwithstanding variations in social communication development.

The comparatively advanced forms and increased frequency of communicative acts by children in the early and late first words/word combinations phases also help to explain the unexpected finding that their parents used a significantly larger proportion of follow-in directives for language than did those of children in preverbal phase. Recognizing that their child had acquired words or phrases, parents may have been more likely to invite their child to use more of them. Interestingly, parents of children in the preverbal phase were observed to use a significantly larger proportion of follow-in directives for *action* than did those in the later language phases. One direction for future research may be to examine associations between follow-in directives for action and child engagement, motor, or play skills, as statistically significant relationships with communication and language were not identified in this study.

Finally, it is important to consider the value of all types of synchronous PVR. Responsiveness to a child’s focus of attention has been shown to be important in earlier stages of language learning, while expansions and follow-in directives may be more strongly related to language development for toddlers who have acquired single words or word combinations (Haebig et al., 2013a, 2013b; Hoff & Naigles, 2002; Vallotton et al., 2017). We observed a significant, negative relationship between follow-in nonverbal comments and follow-in directives for action and prospective language skills; however, use of this PVR type may still serve to support sustained child social attention and engagement in interaction for children communicating in the preverbal phase.

### Concurrent and Prospective Relationships

Finally, our prediction that parents’ use of linguistic mapping, expansions, and follow-in directives would be related to concurrent social communication and prospective receptive and expressive language skills, was partially supported. All three types of synchronous PVR were observed to have statistically significant correlations with the CSBS total score and MSEL verbal DQ. When we entered these variables into our follow-up hierarchical regressions, they each contributed significantly to the concurrent model. In the prospective model, expansions and follow-in directives for language explained significant variance in language outcomes, while linguistic mapping did not. These findings are consistent with earlier studies that included older children with autism (Haebig et al., 2013a; McDuffie & Yoder, 2010; cf. Smith et al., 2022). Follow-in directives and comments, although one type of PVR is more demanding than the other both offer augmented language input to the child. Each has the potential to facilitate a mapping between words and people, objects, or events. Synchronous, directive language that encourages a child to answer a question or expand their utterance may also support sustained social attention in young children with DD and autism who have difficulty shifting focus between objects and communication partners. Some researchers have endorsed the exclusion of directive utterances from studies of PVR based on results suggesting that demanding language may inhibit child communication and engagement. However, our findings concur with previous work in this area supporting parents’ use of both types of PVR—responsive as well as directive—as they respond to their child’s focus of attention and communicative acts (Bottema-Beutel et al., 2021; Haebig et al., 2013a; Walton & Ingersoll, 2015).

### Clinical Implications

Taking our results into account, intervention targets that aim to increase opportunities to embed PVR in the home environment may be refined and tested in future experimental research (e.g., Davis et al., 2022; McDaniel et al., 2021). First, the synchronous PVR parents are already providing, both responsive and directive in form, should be encouraged and enhanced. In this study, parents of children across groups used expansions only 1% of the time and follow-in directives for language 17% of the time. In contrast, 37% of responses were follow-in nonverbal comments. Given the significant contributions of expansions and follow-in directives for language to developmental outcomes, parents of children with autism and DD may be encouraged to map a word or short phrase onto their child’s communicative act, even when their intentions are not entirely clear, or they are not yet using many single words. For example, parents could offer an expansion in response to a preverbal intentional communicative act. If the child points to indicate a dog and makes a sound, the adult could expand with, “A dog. The dog says, ‘ruff!’” Another strategy might be to combine PVR types by providing an expansion, then a follow-in directive for language, in the same response (e.g., Child says, “Doggie,” and the adult responds, “It’s a doggie! Can you say, ‘Ruff ruff, doggie!’”). Until these strategies are empirically evaluated, however, early intervention providers must exercise care in making these recommendations. The benefits of encouraging parents to provide language input to preverbal toddlers with autism and DD remain under investigation.

By and large, results of observational research studies like this one indicate that parents of children with and without autism and DD, on average, may be exceptionally responsive to their children’s attentional focus and communication. A growing number of studies of parent-implemented early intervention models indicate that significant changes in PVR are related to improved child social communication and language skills (e.g., Brian et al., 2022; Wetherby et al., 2014); however, others report increased PVR without corresponding effects on child outcomes (Carter et al., 2011; Rogers et al., 2012; Watson et al., 2017). Our findings suggest that stronger child expressive language promotes parental use of expansions and follow-in directives for language. Consequently, there is a need for further inquiry into strategies that may increase child language and joint attention, beyond providing synchronous PVR, that can be infused into parent-implemented intervention models for young children with autism and DD.

### Limitations and Future Directions

Limitations to this study are important to note. First, our DD and TD groups were smaller than the group of children with autism; therefore, we conservatively controlled for type I error in our analyses. Next, families were given a standard set of instructions for the home observation to include 5–6 different activities; however, they chose their activities and determined how long they spent in each within the hour. This introduced a lack of control over activity and participant variables that should be investigated further. The structure of our data did not allow us to account for variability in patterns of PVR that may have been related to the presence of mothers, fathers, or additional children in this study. Furthermore, although we examined patterns of PVR over a longer period than many previous studies, the potential for an observer effect on parents’ behavior must be considered. A future direction of this research is to record multiple sessions and couple observational coding methods with automated analyses (e.g., Lopez et al., 2020). Another direction will be to use sequential analytic techniques to examine temporal associations between child communication and parental responses as they unfold in time (Bottema-Beutel et al., 2021). A potential moderating variable that may have altered the prospective relationships documented in this study was the inclusion of 39 children with autism who participated in a randomized controlled trial (RCT) of the Early Social Interaction intervention model (Wetherby et al., 2014). The home observation was taken at baseline, before enrollment in the RCT. Additionally, we removed these participants from analyses, and correlation coefficients remained consistent in effect size. A related limitation is that information about intervention services delivered to children with autism and DD, and the training and coaching relative to PVR their parents may have received, was not available. Finally, additional studies of PVR are needed before results may be generalized to the larger population of toddlers with autism, DD, and TD.

## Conclusion

Research evidence thus far indicates that parent verbal responsiveness is related to a range of positive developmental outcomes in children with and without developmental delays and autism. Observation of parent–child interaction in the home environment provides useful information about children’s strengths and needs and the importance of contingent, synchronous parent verbal responsiveness. Results of this study contribute to our growing understanding of factors in the learning environment that impact social communication and language skills in children with DD and autism and provide directions for future experimental intervention research.

### Supplementary Information

Below is the link to the electronic supplementary material.

Supplementary file1 (DOCX 617 kb)
