# Supplementary material for: Verbal Responsiveness in Parents of Toddlers With and Without Autism During a Home Observation
Source: J Autism Dev Disord. 2023 May 12;54(7):2440–53. doi: 10.1007/s10803-023-05935-6 (PMC11286669; doi:10.1007/s10803-023-05935-6)

*Supplemental Table S1*

Participant Developmental Characteristics

Reprinted with permission of the authors [blinded].


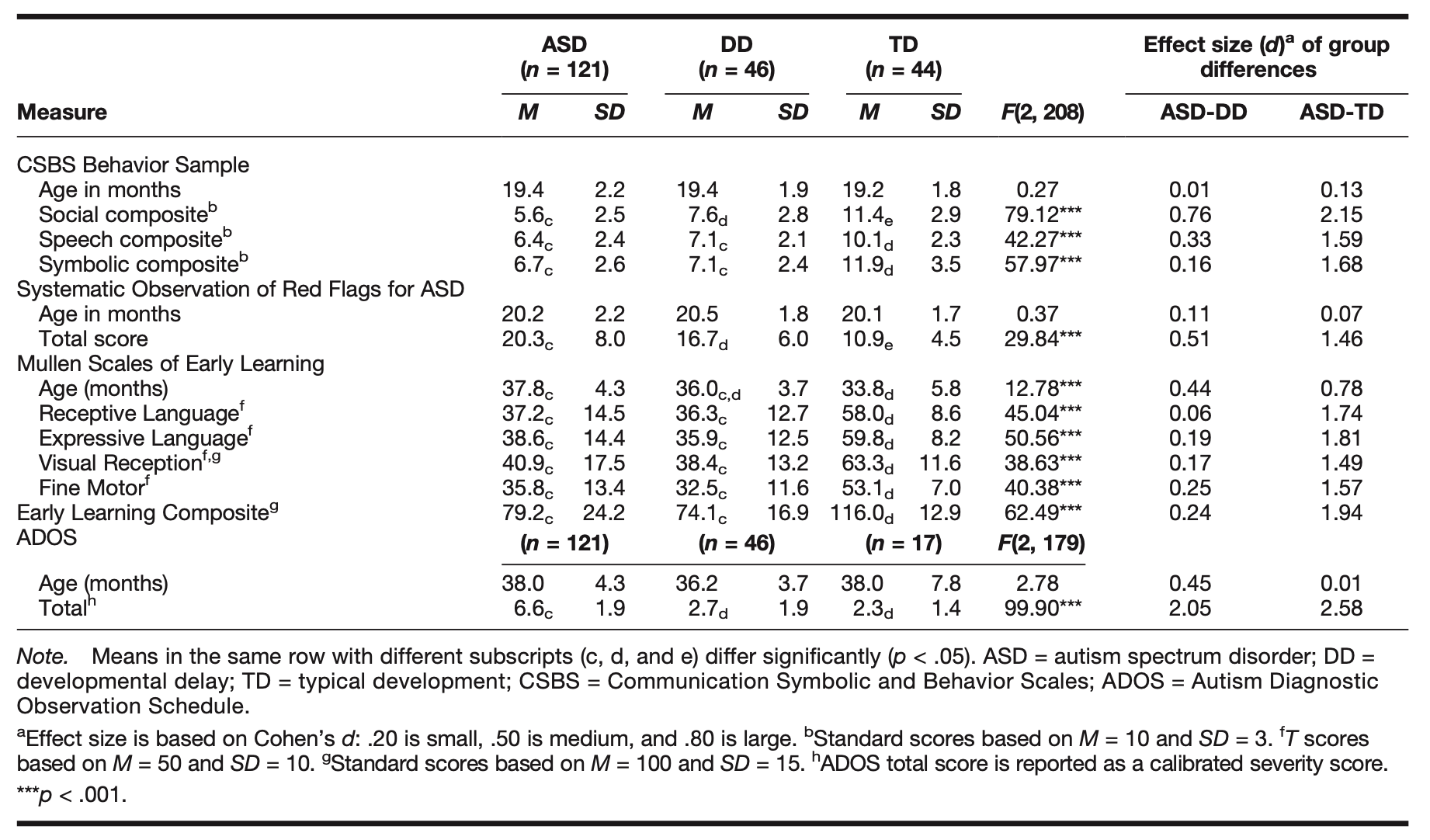


*Supplemental Table S2*

Participant Demographic Characteristics

Reprinted with permission of the authors [blinded].


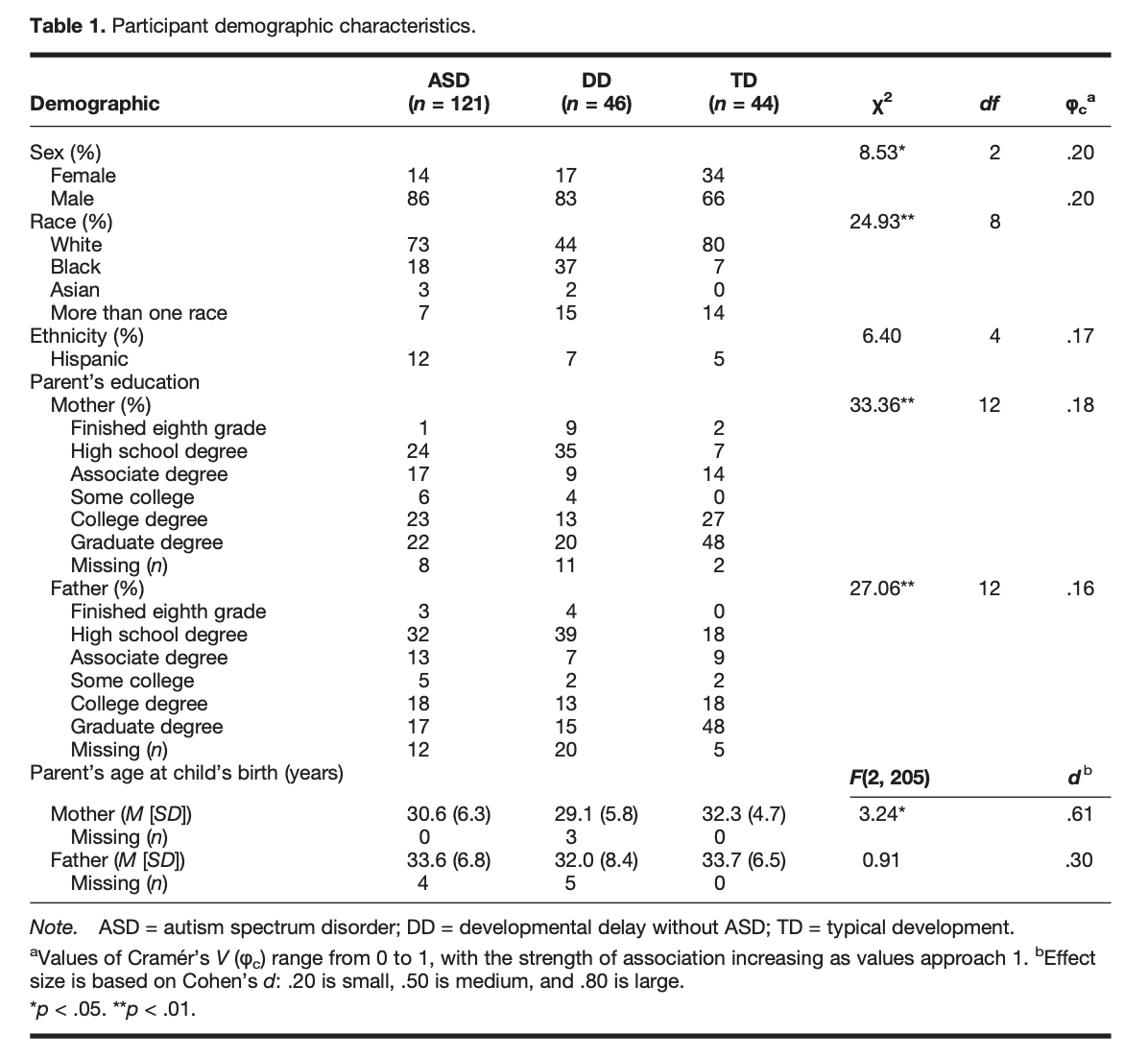

Supplement: Supplementary file 1 — Supplementary file1 (DOCX 617 kb) [file 10803_2023_5935_MOESM1_ESM.docx]
